# Mean platelet volume and cardiovascular outcomes in patients with coronary heart disease: A meta-analysis of prospective studies

**DOI:** 10.1097/MD.0000000000041818

**Published:** 2026-01-23

**Authors:** Qing-Chuan Wang, Ye Chen, Chun-Li Guan, Wan-Ke Lu

**Affiliations:** a Department of General Pratice, Shenzhen Longhua District Central Hospital, Shenzhen, China.

**Keywords:** all-cause mortality, coronary heart disease, major adverse cardiac events, mean platelet volume, meta-analysis

## Abstract

**Background:**

While there is evidence suggesting that increased mean platelet volume (MPV) is linked to adverse cardiac outcomes in patients with acute coronary syndrome, the association between MPV and clinical outcomes in patients with coronary heart disease (CHD) remains a topic of debate. This meta-analysis focuses on comprehensively reviewing existing research regarding the association between MPV levels and the prognosis of patients with CHD.

**Methods:**

We included all relevant prospective studies carried out before January 31, 2024. The cutoff values, which also defined high/low-MPV levels, had transformed continuous variables into a dichotomous variables. The results of these studies reported hazard ratios (HRs) for all-cause mortality or incidence of major adverse cardiac events (MACEs).

**Results:**

A total of 10 prospective studies, including 11,647 patients with CHD, were enrolled in this meta-analysis. The pooled HR for long-term all-cause mortality was 1.06 (95% confidence interval [CI], 0.89–1.27), with no statistically significant association between increased MPV and long-term all-cause mortality in patients with CHD. Increased MPV level was associated with a significantly increased risk of short-term all-cause mortality (HR, 2.10 [95% CI, 1.34–3.29]; *P* < .05). For the incidence of long-term MACEs, the pooled HR for long-term MACE occurrence was 1.14 (95% CI, 1.02–1.28), showing a statistically significant correlation with increased MPV in patients with CHD.

**Conclusion:**

This present meta-analysis showed that an increased MPV was associated with an increased risk of short-term all-cause mortality and long-term MACEs in patients with CHD. No association was found between elevated MPV levels and the incidence of long-term mortality in patients with CHD.

## 1. Introduction

Coronary heart disease (CHD) has become the most important cause of noninfectious disease deaths worldwide,^[1]^ posing a serious challenge to the quality of human life.^[2]^ Currently, there are many known risk factors for CHD, such as hypertension, diabetes mellitus, smoking, and obesity, which may affect the course of CHD.^[3–6]^ However, some patients with CHD with poor prognosis did not have the risk factors mentioned above.

Platelets play important roles in the pathogenesis of atherosclerosis and arterial thrombosis.^[7,8]^ Mean platelet volume (MPV) is a commonly used parameter for predicting functional changes and platelet activation by estimating the average size of platelets. MPV can also be an indicator of platelet production, which can lead to increased platelet adhesion and aggregation, eventually resulting in vascular embolism. In addition, MPV is simple, inexpensive and widely available based on complete blood count. Although elevated MPV levels have been reported to be related to cerebrovascular disease, CHD, and acute myocardial infarction (AMI),^[9–11]^ some studies have reported that elevated MPV may not be considered a risk factor for CHD.^[12–15]^ Wada et al^[16]^ showed that a low MPV was associated with poor clinical outcomes among patients with CHD. Therefore, the association between elevated MPV levels and poor clinical outcomes in patients with CHD remains uncertain. We undertook this meta-analysis based on relevant and available prospective cohort studies to investigate the effect of MPV levels in patients with CHD.

## 2. Methods

This meta-analysis was conducted according to the Preferred Reporting Items for Systematic Reviews and Meta-Analyses (PRISMA),^[17]^ and Meta-analysis of Observational Studies in Epidemiology statements.^[18]^ The meta-analysis was prospectively registered in PROSPERO (CRD 42023404388). Our meta-analysis was based on published articles, therefore, there was no need for ethical approval.

### 2.1. Search strategy

Two researchers independently extracted published articles in PubMed, Web of Science, EMBASE, CISCOM and the Cochrane Library, and systematically searched the published articles up to March 31, 2024, for the impact of MPV levels in CHD patients. The search terms were as follows: “mean platelet volume,” “MPV,” “myocardial, coronary,” “coronary arteriosclerosis,” “coronary artery disease,” “ACS,” “coronary heart disease,” “ CHD,” “major adverse cardiac events,” “MACE,” “all-cause mortality,” “death,” “hazard ratio,” and “HR.” The language of the studies was limited to English. We also searched the bibliographies of relevant studies to identify all potentially relevant studies. Reference lists of previous systematic reviews and all selected studies were explored again to identify additional eligible studies.

### 2.2. Selection of studies

Inclusion criteria of this meta-analysis were prospective studies published in English, with the study population restricted to patients with CHD (including unstable angina, ST-segment–elevation myocardial infarction, non–ST-segment–elevation myocardial infarction (NSTEMI), stable angina, ischemic cardiomyopathy and latent CHD), and the included studies had full text available. The results of the included studies need to consider the hazard ratio (HR) and its 95% confidence intervals (CI) to assess the association between MPV levels and the prognosis of patients with CHD, from which we could extract information about the HR. The endpoints were MACEs (the composite of cardiovascular mortality, reinfarction, cardiogenic shock, angina, ventricular fibrillation, stent thrombosis, revascularization, stroke and left-ventricular dysfunction, major bleeding and heart failure) and all-cause mortality.

We excluded any duplicate records, retrospective studies, and literature reviews. We conducted a preliminary screening of abstracts and titles of the studies to determine their relevance, categorizing them as either excluded or in need of further assessment. Any disagreements among the researchers were resolved through collaborative discussions or by seeking the input of a third researcher.

### 2.3. Data extraction and quality assessment

Two independent investigators who were trained and proficient in medical statistics participated in extracting data from the included studies to enhance credibility, reduce selection bias and reinforce credibility. Disagreements were resolved by discussion between the 2 investigators. The following items were extracted from the included studies for the meta-analysis: first author’s name, year of publication, participants’ characteristics, study design, average follow-up duration, cutoff value of MPV level, clinical outcomes (long-term mortality, long-term MACEs and short-term mortality), HR and its 95% CI, adjusted covariates in the multiple factor regression analysis, and total numbers of individuals. We extracted higher and lower groups with a range of MPV to assess the association between MPV level and prognosis of patients with CHD.

All the enrolled criteria were assessed by 2 independent researchers according to the Newcastle-Ottawa Scale (NOS),^[19]^ which contains 3 dimensions: representativeness of the participants, comparability between exposed and nonexposed groups, and measurability of outcome. The NOS ranges from 0 to 9 stars. Generally, 7 to 9 points belong to high score and high-quality research, 4 to 6 points belong to medium quality, and ≤ 4 points belong to low-quality research.

### 2.4. Ethics

Because no original clinical data were collected or utilized in previously published studies, ethical approval was not necessary for this meta-analysis.

### 2.5. Statistical analysis

The extracted data were analyzed by STATA 16.0. Clinical endpoint events were calculated using pooled HRs among studies. Heterogeneity level among the enrolled studies was assessed using the Cochran *Q* test (*P* < .10, implying that heterogeneity was statistically significant) and *I*^2^ statistic (*I*^2^ < 25%, no heterogeneity; 25%≤ *I*^2^ ≤ 50%, mild heterogeneity; 50%<*I*^2^ ≤ 75%, moderate heterogeneity; and *I*^2^ > 75%, severe heterogeneity). Inconsistencies across studies were quantified using *I*^2^ statistics. When heterogeneity between studies was statistically significant, we chose the random-effects model; otherwise, we chose the fixed-effects model. When the heterogeneity was too large, sensitivity analysis was conducted to further analyze the source of potential heterogeneity, in which each study, one at a time, was deleted to see whether the pooled HR would be significantly affected. Publication bias was assessed when the number of studies was enrolled.

The data were independently input into the software by 2 investigators to ensure that the results were credible and accurate. Statistical significance was set at *P* < .05.

## 3. Results

### 3.1. Selection of studies

After a total and comprehensive search, we identified 878 potentially relevant articles. Of these, 65 were excluded after screening their titles and abstracts. In addition, we excluded 5 retrospective studies, 9 reviews, 11 studies due to missing follow-up data, and 10 studies due to the absence of mortality or MACEs. Finally, 10 prospective cohort studies were included in the meta-analysis. A detailed search strategy is shown in the flow diagram (PRISMA Flow Diagram).

### 3.2. Characteristics of studies

We identified 10 prospective studies^[15,16,20–27]^ aimed at investigating the relationship between MPV and clinical outcomes (long-term mortality, long-term MACEs and short-term mortality) in patients with CHD (including unstable angina, ST-segment–elevation myocardial infarction, NSTEMI, stable angina, ischemic cardiomyopathy and latent CHD). Ten studies were published between 2005 and 2020, and 10 studies with 11,647 participants were included in the final analysis. Of these, 2 studies^[25,27]^ were conducted in China and two^[15,21]^ in Spain. 1 study each was conducted in Japan,^[16]^ Turkey,^[23]^ Italy,^[22]^ Poland,^[20]^ India^[24]^ and Greece.^[26]^ The sample sizes in these studies varied from 104 to 4293. The mean age of the participants in the individual studies ranged from 56 to 76 years. The duration of follow-up for these studies ranged from 1 to 67.2 months (shown in Table 1 and Table S1, Supplemental Digital Content, http://links.lww.com/MD/O516).

**Table 1:** Main characteristics of the included studies in the meta-analysis.

Author, year	Country	Sample	Mean age (yr)	Participants	Cutoff value	End point	Follow-up (mo)
Huczek et al 2005^[20]^	Poland	388	60 ± 11.3	STEMI	10.3	All-cause mortality	6
Estévez-Loureiro et al 2009^[21]^	Spain	617	63 ± 12	STEMI	8.95	All-cause mortality	1
Taglieri et al 2011^[22]^	Italy	1041	76 ± 7.5	ACS	8.9	All-cause mortality/MACEs	12
Dogan et al 2012^[23]^	Turkey	344	62.2 ± 7.8	ACS	9.9	MACEs	12
López-Cuenca et al 2012^[15]^	Spain	329	67.3 ± 12.3	ACS	11.0	MACEs	6
Ranjith et al 2016^[24]^	India	1206	56 ± 11.1	AMI	11.0	MACEs	12
Lai et al 2016^[25]^	China	453	56.2 ± 8.4	STEMI	9.85	All-cause mortality	1
Wada et al 2018^[16]^	Japan	2872	67 ± 10.0	CHD	10.9	All-cause mortality/MACEs	67.2
Vogiatzis et al 2019^[26]^	Greece	104	64.2 ± 11.1	ACS	7.5	MACEs	1
Jiang et al 2020^[27]^	China	4293	58 ± 10.0	CHD	10.5	All-cause mortality/MACEs	24

ACS = acute coronary syndromes, CHD = coronary heart disease, MACE = major adverse cardiac event, STEMI = ST-segment–elevation acute coronary syndrome.

The overall NOS scores of the included studies ranged from 7 to 9 (shown in Table 2). All the included studies were limited to prospective studies in patients with CHD. The primary endpoints reported in the 10 studies varied, including all-cause mortality and MACEs. Four studies^[16,20,22,27]^ provided information about long-term mortality, 6 studies^[15,16,22–24,27]^ provided information about the incidence of MACEs, 2 studies^[21,25]^ were followed-up for 30 days to investigate the association between admission MPV and in-hospital mortality, and only 1 study^[26]^ was followed-up for 30 days to assess the association of admission MPV with in-hospital MACEs. Regarding the exposure factor, the MPV level was divided into higher and lower MPV groups. All the enrolled studies provided HRs calculated using the multiple factor regression model. More detailed information on the included studies is shown in Table 1.

**Table 2:** Newcastle-Ottawa Scale.

Study	Selection				Comparability		Exposure			Quality
	Case definition	Representativeness of the cases	Selection of Controls	Definition of Controls	Comparability: Basic Factors	Comparability: Additional actors	Ascertainment of exposure	Same method of ascertainment for cases and controls	nonResponse rate: same rate for both groups	
Huczek et al 2005^[20^^]^	★	★	★	★		★	★	★	★	**8**
Estévez-Loureiro et al 2009^[21]^	★	★	★	★		★	★	★	★	**8**
Taglieri et al 2011^[22]^	★	★	★	★	★	★	★	★	★	**9**
Dogan et al 2012^[23]^	★	★	★	★		★	★	★	★	**8**
López-Cuenca et al 2012^[15]^	★	★	★	★		★	★	★	★	**8**
Ranjith et al 2016^[24]^	★	★	★	★		★	★	★	★	**8**
Lai et al 2016^[25]^	★	★	★	★	★	★	★	★	★	**9**
Wada et al 2018^[16]^	★	★	★	★	★	★	★	★	★	**9**
Vogiatzis et al 2019^[26]^	★	★	★	★			★	★	★	**7**
Jiang et al 2020^[27]^	★	★	★	★	★	★	★	★	★	**9**

### 3.3. Association between MPV level and long-/short-term all-cause mortality in patients with CHD

**Figure 1.**
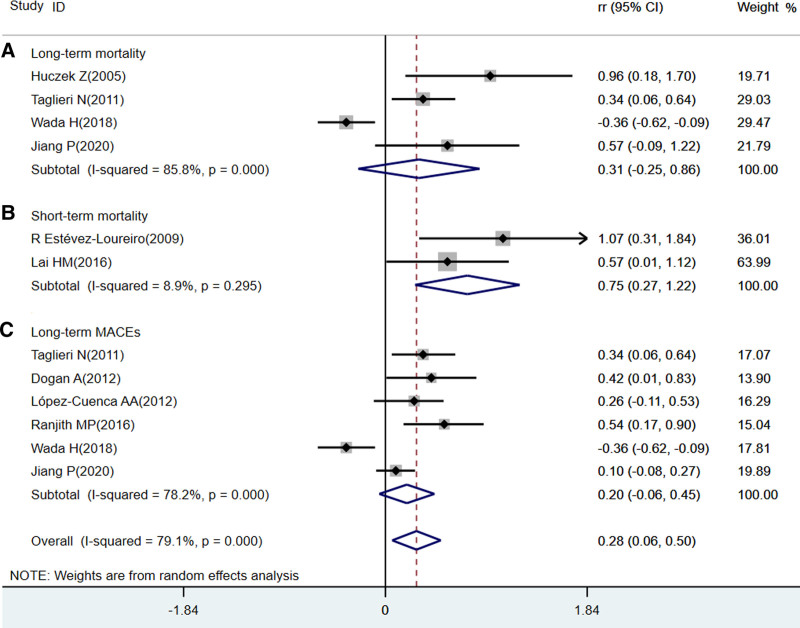
Forest plot of the association between MPV and outcomes in patients with CHD. (A) The association between high serum MPV levels and the incidence of long-term all-cause mortality. (B) The association between high serum MPV levels and the incidence of short-term all-cause mortality. (C) The association between high serum MPV levels and the incidence of long-term MACEs. CHD=coronary heart disease, CI=confidence interval, HR=hazard ratio, MACE=major adverse cardiac event, MPV=mean platelet volume.

**Figure 2.**
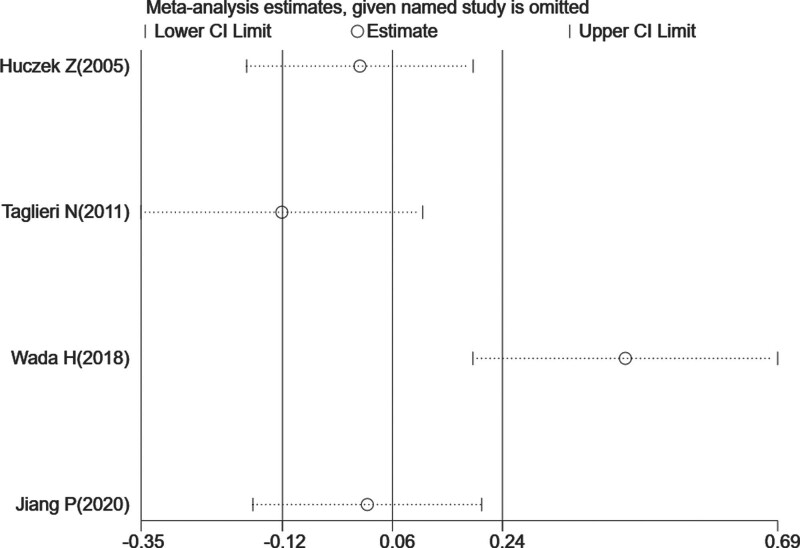
Results of sensitivity analysis for long-term mortality. The middle vertical line indicates the pooled HR of 4 studies, and the 2 vertical lines represent the 95% CI values. Every hollow round indicates the pooled HR when the left study was omitted in a meta-analysis. CI=confidence interval, HR=hazard ratio.

Four studies^[16,20,22,27]^ focused on long-term mortality analysis, 3^[16,20,22,27]^ of which considered all-cause mortality and MACEs as outcome events. Compared with the low-MPV group, the pooled HR for long-term mortality was 1.06 (95% CI, 0.89–1.27), showing no statistically significant association between increased MPV and long-term all-cause mortality in patients with CHD. The *P* value of the Cochran Q-statistic was ≤ .01, and *I^2^* was 85.8% (shown in Fig. 1). Sensitivity analysis was conducted because the heterogeneity among the 4 studies was statistically significant. The sensitivity analysis showed that when the study conducted by Wada et al^[16]^ was removed, the heterogeneity changed significantly. We performed a subgroup analysis based on geographic locations (Europe and Asia), study population (CHD and ACS), sample size (<2000 and ≥2000), age (<62 years old and ≥62 years old) and duration of follow-up (<3 years and ≥3 years; shown in Table 3). In the subgroup of duration of follow-up (<3 years), *I*^2^ decreased to 14.0%, which showed that the subgroup of follow-up duration was a significant source of heterogeneity (shown in Figure 2).

**Table 3:** The association between the MPV and long-time morality according to different subgroups

Subgroup		Study (no.)	*I*^2^ (%)	*P (I*^2^)	HR (95% CI)	*P* (HR)
Geographic locations	Europe	2	53.9	.141	1.52 (1.16–2.00)	.002
	Asia	2	84.6	.011	0.79 (0.62–1.01)	.062
Study population	CHD	2	84.6	.011	0.79 (0.62–1.01)	.062
	ACS	2	53.9	.141	1.52 (1.16–2.00)	.002
Sample size	<2000	2	53.9	.141	1.52 (1.16–2.00)	.002
	≥2000	2	84.6	.011	0.79 (0.62–1.01)	.062
Duration of follow-up	<3 years	3	14	.313	1.56 (1.21–2.00)	.001
	≥3 years	1	0	–	0.7 (0.54–0.91)	.007
Age	<62 years old	2	0	.446	2.08 (1.26–3.42)	.004
	≥62 years old	2	91.9	0	0.96 (0.79–1.16)	.668

CI = confidence interval, HR = hazard ratio, MPV = mean platelet volume.

Two studies^[21,25]^ considered short-term mortality as the outcome. The elevated MPV group had a significantly increased risk of short-term all-cause mortality (HR, 2.10 [95% CI, 1.34–3.29]; *P* < .05), compared with the low-MPV group. The *P* value of the Cochran Q-statistic was 0.30, and *I^2^* was 8.9% (shown in Fig. 1). Sensitivity analysis was not conducted because the heterogeneity was too small.

The number of studies enrolled, with endpoint events being long-/short-term mortalities, was <10, so we did not run the publication bias statistics because the bias effect might be overestimated.

### 3.4. Association between MPV level and long-term MACEs in patients with CHD

**Figure 3.**
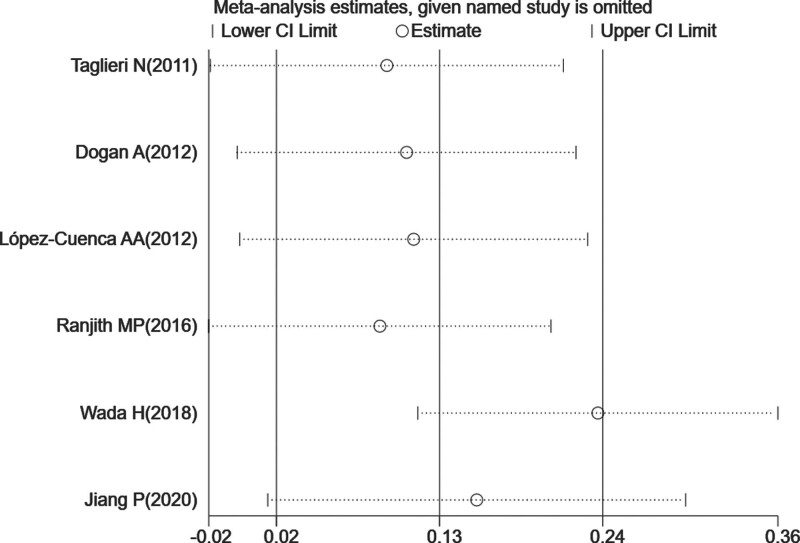
Results of sensitivity analysis for long-term MACEs. The middle vertical line indicates the pooled HR of 6 studies, and the 2 vertical lines represent the 95% CI values. Every hollow round indicates the pooled HR when the left study was omitted in a meta-analysis. CI=confidence interval, HR=hazard ratio, MACE=major adverse cardiac event.

Three^[15,23,24]^ of the 6 enrolled studies regarded the incidence of long-term MACEs as an endpoint event. For the incidence of long-term MACEs, the pooled HR was 1.14 (95% CI, 1.02–1.28), showing a statistically significant association between increased MPV and the incidence of long-term MACEs in patients with CHD, compared with the low-MPV group. The *P* value of the Cochran Q-statistic was ≤ .01, and *I*^2^ was 78.2%. A sensitivity analysis was performed due to the statistically significant heterogeneity evident among the 4 studies (shown in Fig. 2). The sensitivity analysis revealed that, after excluding the study by Wada et al,^[26]^ the largest mean difference between the 2 groups occurred where a high MPV level was significantly linked to a higher long-term MACE risk in patients with CHD, in comparison to the low-MPV group (shown in Fig. 3).

## 4. Discussion

Globally, approximately one-half of noncommunicable disease death rates account for cardiovascular diseases, and CHD has become an increasingly serious public health problem.^[1]^ MPV is a simple, cost-effective and practical method that has been reported to be involved in the course of CHD because large platelets have higher blood thrombogenesis activity.^[28]^ However, the association between MPV levels and endpoint events in patients with CHD remains a topic of dispute.

MPV is a simple and, widely available metric that is determined using ordinary automated analyzers.^[29]^ An elevated MPV was associated with large platelets and greater prothrombotic activity.^[30]^ A recent meta-analysis^[31]^ indicated an association between elevated MPV and long-term mortality in patients with ACS, but not CHD. In addition, a prior meta-analysis^[9]^ showed an association between elevated MPV and cardiovascular events in CHD patients, and a dose-response relationship was also observed in this meta-analysis, however, it did not assess the influence of MPV on the occurrence of all-cause mortality and MACEs in patients with CHD, which was analyzed over a decade ago. Therefore, our meta-analysis combined all the latest studies to fill the large gaps in knowledge regarding the value of MPV on CHD progression.

Our meta-analysis showed that high MPV levels were associated with an increased risk of short-term all-cause mortality (HR, 2.10 [95% CI, 1.34–3.29]; *P* < .05) and long-term MACEs (HR, 1.14 [95% CI, 1.02–1.28]; *P* < .05), compared with the low-MPV level group (shown in Fig. 1), which was different from previous research. Supel et al^[32]^ showed that MPV may not be a predictor of poor in-hospital outcomes in patients with CHD. Our meta-analysis indicated that no association between elevated MPV levels and the incidence of long-term mortality was found in patients with CHD (HR, 1.06 [95% CI, 0.89–1.27]; *P* = .518), compared with the low-MPV level group (shown in Fig. 1). However, some cohort studies^[33,34]^showed conflicting results and reported that elevated MPV was a significant risk factor for 2-year cardiac mortality, and elevated MPV did not predict in-hospital mortality in the NSTEMI group. Nevertheless, some findings from cohort studies^[35]^ support the possible value of MPV, as a prognostic biomarker, for predicting future clinical endpoint events in patients with CHD.

Previous studies have predominantly focused on specific endpoints. For instance, the study by Chen et al^[36]^ evaluated short-term and long-term mortality in patients with AMI but did not assess MACEs. Similarly, the meta-analysis by Chu et al^[37]^ examined mortality and restenosis following coronary angioplasty in patients with AMI, omitting MACEs. Another meta-analysis by Galimzhanov et al^[31]^ included 41 studies with 33,443 patients with ACS, primarily focusing on long-term mortality and MACEs but neglecting short-term mortality. The most recent systematic review by Galimzhanov et al^[38]^ included 52 studies with 47,066 patients with CAD, focusing mainly on long-term mortality without evaluating short-term mortality or MACEs. In contrast, our meta-analysis stands out by providing a comprehensive evaluation of MPV’s prognostic significance in CHD. By including both short-term and long-term mortality as well as MACEs, our meta-analysis offers a more complete picture of how MPV levels can influence various clinical outcomes in patients with CHD. Moreover, our study innovates by conducting detailed stratified analyses of MPV levels.

In addition, when the heterogeneity of the enrolled studies was analyzed, we found significant heterogeneity (*I*^2^ = 85.8%, *P*_heterogeneity_ < .001). We then performed subgroup analysis according to geographic location (Europe and Asia), study population (CHD and ACS), age (<62 years old and ≥62 years old), sample size (<2000 and ≥2000) and duration of follow-up (<3 years and ≥3 years). In the subgroup for duration of follow-up (<3 years), *I*^2^ decreased to 14.0%, which showed that the subgroup for duration of follow-up might be a significant source of heterogeneity (shown in Table 3). We suspected that several factors, except one, might play a key role in the high heterogeneity because sensitivity analysis might not clearly assess sources of heterogeneity.

Although this meta-analysis was relatively comprehensive and objective, it has a few limitations. First, the number of studies and study sample sizes was small, and only 1 enrolled study^[26]^ reported the HR for in-hospital MACEs. Further research should be conducted to confirm these effects in the future. Second, the variability in individual MPV at different times during a patient’s lifetime is significant. The differing pathophysiology in the patient with CHD population, such as population geography, health care systems and European vs non-European countries limits the certainty of the conclusions. Third, the large heterogeneity among the studies might affect the association between MPV levels and the prognosis of patients with CHD, especially in the long-term mortality group, and more large-scale prospective studies should be conducted in the near future. Finally, 9 studies did not have an average follow-up period ≥ 2 years, while only 1 study^[26]^ had just a 5.6-year follow-up, which may affect the assessment of long-term prognosis. In addition, our meta-analysis exclusively included prospective studies. While this approach reduces the biases that may arise from retrospective studies, it also limits the diversity of the research designs included.

## 5. Conclusion

In patients with CHD, high serum levels of MPV were associated with an increased risk of long-term MACEs and short-term mortality. No association between high serum MPV levels and the incidence of long-term mortality was found in patients with CHD. Owing to the limitations mentioned above, more prospective and controlled studies need to be conducted to make the results more comparable and reliable.

## Acknowledgments

The authors thank all participants who contributed to this meta-analysis.

## Author contributions

**Data curation:** Qing-Chuan Wang.

**Formal analysis:** Qing-Chuan Wang.

**Writing – original draft:** Qing-Chuan Wang.

**Supervision:** Chunli Guan.

**Validation:** Chunli Guan.

**Methodology:** Ye Chen

**Resources:** Ye Chen.

**Visualization:** Wan-Ke Lu.

**Writing – review & editing:** Wan-Ke Lu.

## Supplementary Material


