# Supplementary material for: Mean platelet volume and cardiovascular outcomes in patients with coronary heart disease: A meta-analysis of prospective studies
Source: Medicine (Baltimore). 2026 Jan 23;105(4):e41818. doi: 10.1097/MD.0000000000041818 (PMC12851692; doi:10.1097/MD.0000000000041818)
Supplement: Supplementary file 1 [file medi-105-e41818-s001.doc]

Supplementary table 1. The definition of short- and long- term outcomes

| Author, year | Country | Sample | short- term outcomes | long- term outcomes | The endpoint |
| --- | --- | --- | --- | --- | --- |
| Huczek Z et al, 2005^[20]^ | Poland | 388 | - | 6-month mortality | 6-month mortality |
| Estévez-Loureiro R et al, 2009^[21]^ | Spain | 617 | 30-day mortality | - | 30-day mortality |
| Taglieri N et al, 2011^[22]^ | Italy | 1041 | - | 12-month mortality/MACEs | Cardiovascular death and re-myocardial infarction,  Individual cardiovascular death and re-MI, major bleeding, stroke |
| Dogan A et al, 2012^[23]^ | Turkey | 344 | - | 12-month MACEs | The composite of cardiac death, nonfatal MI, recurrent angina, or hospitalization for heart failure |
| López-Cuenca AA et al,2012^[15]^ | Spain | 329 | - | 6-month MACEs | Cardiovascular death and new ACS |
| Ranjith MP et al, 2016^[24]^ | India | 1206 | - | 12-month MACEs | Cardiovascular mortality, stroke, non-fatal MI and cardiac failure |
| Lai HM et al, 2016^[25]^ | China | 453 | 30-day all-cause mortality | - | 30-day all-cause mortality |
| Wada H et al, 2018^[16]^ | Japan | 2872 | - | mortality/MACEs^*^ | All-case death and non-fatal MI |
| Vogiatzis I et al, 2019^[26]^ | Greece | 104 | 30-day MACEs | - | Death, stent thrombosis, reinfarction, ventricular fibrillation, cardiogenic shock, angina, left-ventricular dysfunction and stroke |
| Jiang P et al, 2020^[27]^ | China | 4293 | - | 24-month mortality/MACEs | Death, cardiovascular death, MI, stent thrombosis, revascularization and stroke |

^*^Median follow-up of 5.6 years. ACS: acute coronary syndromes, MI: myocardial infarct, ACS: acute coronary syndrome, MACEs: major adverse cardiac events.
